# Adjusting for Glycemic Control in Assessing the Relationship Between Age-Related Macular Degeneration and Diabetic Retinopathy

**DOI:** 10.7759/cureus.71479

**Published:** 2024-10-14

**Authors:** Michael Wolek, Brian Wollocko, Deborah M Li, Jahnvi Bansal, Nimra Ghani, Michael Mackey, Khurram Chaudhary

**Affiliations:** 1 Ophthalmology, University Hospitals Cleveland Medical Center, Cleveland, USA; 2 Ophthalmology, State University of New York Downstate Health Sciences University, New York, USA; 3 Ophthalmology, Stony Brook University, Stony Brook, USA; 4 Anesthesiology, Westchester Medical Center, Valhalla, USA; 5 Internal Medicine, Stony Brook University, Stony Brook, USA

**Keywords:** age-related macular degeneration (amd), diabetes type ii, diabetic retinopathy, glycemic control and diabetes, hemoglobin a1c (hba1c), risk factor investigation

## Abstract

Purpose

Studies regarding the relationship between age-related macular degeneration (AMD) and diabetic retinopathy (DR) conflict: while some support that AMD is protective against DR, others find the opposite. The mechanism by which AMD may protect against DR is unclear. We sought to assess the association between AMD and DR when controlling for glycemic control in patients with diabetes mellitus (DM) type II.

Methods

We identified 461 unique patients over 55 years old with a diagnosis of DM type II seen in our academic retina clinic in Stony Brook, New York between 12/31/2019 and 12/31/2020. Data were manually extracted and the population was split based on the presence of AMD diagnosis. Multivariate regression analyses were then performed comparing the prevalence of DR between groups while controlling for A1c. Secondary endpoints included demographic differences and smoking status.

Results

Among the 461 patients, 118 (25.6%) had a diagnosis of AMD. Compared to patients without AMD, patients with AMD were older (69 vs. 66; OR 1.05; p=0.005) and less likely to have DR (37.3% vs. 59.2%; OR 0.35; p<0.001). There was no difference in average A1c between groups.

Conclusion

This is the first reported study assessing the relationship between AMD and DR while controlling for A1c. In our population, diagnosis of AMD was associated with significantly reduced odds of having DR. As AMD and DR appear to be related even after holding A1c constant, researchers should use caution when using DR as a surrogate for diabetic control in the context of AMD.

## Introduction

Accounting for 8.7% of blindness worldwide, age-related macular degeneration (AMD) is the leading cause of blindness in developed countries. This disease is closely associated with age and poses a substantial global burden expected to grow as the population continues to age [1]. Many studies have identified risk factors for AMD, including increased age, previous cataract surgery, current smoking, family history of AMD, and serum cholesterol levels [2,3].

Our understanding of the pathogenesis of AMD is actively being developed and features the accumulation of extracellular debris, the reduction of choroid thickness, and alterations in the thickness and permeability of Bruch’s membrane. These changes lead to atrophy of the retinal pigment epithelium with drusen deposition in the nonexudative form and choroidal neovascularization in the exudative form [4,5]. 

Methods to treat AMD have substantially improved over time. Anti-VEGF therapy has improved the management of late AMD, though treatments for dry AMD remain in development. Dry AMD is often managed with a combination of vitamins and supplements. The large-scale Age-Related Eye Disease Study (AREDS) in 2001 established this common therapy by finding that a supplement mix of antioxidants and zinc may delay AMD progression [6]. However, as the generalizability of this study is unclear and the reproducibility lacking, the efficacy of AREDS remains highly inconclusive [7,8]. Given treatment uncertainty and the immense burden of AMD, further study into the pathogenesis of the disease merits investigation, in the hopes of finding better therapeutic targets.

Diabetic retinopathy (DR) is the most common complication of diabetes mellitus (DM) and affects nearly all of the over 30 different types of cells in the retina [9]. DR involves a complex pathogenesis including chronic inflammation, retinal neurodegeneration, and most notably, microvascular lesions due to hyperglycemia that result in hallmarks of DR: localized microaneurysms, vascular occlusion, and ischemia; vision loss ensues [10]. Despite the prevalence of DR, current therapies remain limited. While intravitreal anti-VEGF therapies currently constitute first-line DR treatment, their long-term efficacy remains subpar [9,11]. As with AMD, the lack of effective DR treatment highlights the need for a comprehensive understanding of the clinical disease pathway in order to develop targeted interventions.

Especially as both AMD and DR are VEGF-mediated, the prevalence and persistence of both diseases have prompted investigations into their relationship. However, recent studies conflict. A retrospective cohort study in Taiwan by He et al. in over 50,000 patients from 1997 to 2012 showed that DR is independently associated with an increased risk of AMD [12]. Another study in Greece by Bourouki et al. suggests that DR may protect against AMD progression [13]. Other studies have shown AMD to be positively associated with DM [14-16].

Given these inconsistencies, our study aimed to investigate the association between AMD and DR in our local population in Stony Brook, New York. We incorporated a smaller sample size, collected hemoglobin A1c levels, and factored in the presence of other risk factors such as current smoking. Our aim was to stratify our findings against these risk factors and the severity of diabetes in each patient to inform us of the relationship between AMD and DR.

## Materials and methods

Data source

Patient identifiers were gathered from billing records at the Stony Brook University ophthalmology clinic in Stony Brook, NY. The study range was from 12/31/2019 through 12/31/2020. The patient list was further limited to only patients seen in the retina clinic with two retina specialists. Finally, patients were limited to those with the International Statistical Classification of Diseases, Tenth Revision (ICD-10) diagnosis codes for DM type II [17]. The presence of ophthalmologic ICD-10 diagnosis codes (Table 1), the examining provider, and demographic data including age and sex were pulled automatically from the ophthalmology billing records. Criteria for AMD and DR included all degrees of active, respective diagnosis. Optical coherence tomography (OCT) was performed on all AMD patients for AMD diagnosis. Patient information including smoking status, presence of an active metformin prescription, and recent hemoglobin A1c value within three months of the appointment date were manually extracted from the medical record. The collective data was then de-identified and scrambled after manual chart review.

**Table 1:** ICD-10 diagnosis codes used to identify patients Diagnosis codes for AMD and DR based on the ICD-10 [17]. AMD, age-related macular degeneration; DR, diabetic retinopathy; ICD-10, International Statistical Classification of Diseases, Tenth Revision

Condition	ICD-10 codes
AMD	H35.3111, H35.3121, H35.3131, H35.3112, H35.3122, H35.3132, H35.3113, H35.3123, H35.3133, H35.3114, H35.3124, H35.3134
DR	E11.3211, E11.3212, E11.3213, E11.3291, E11.3292, E11.3293, E11.3311, E11.3312, E11.3313, E11.3391, E11.3392, E11.3393, E11.3411, E11.3412, E11.3413, E11.3491, E11.3492, E11.3493

As this study is a retrospective chart review, it was determined to be exempt from full approval from the Stony Brook University Institutional Review Board (IRB). Likewise, the IRB waived the need for patient informed consent as all information was de-identified. This study adhered to the guidelines of the Declaration of Helsinki.

Exclusion criteria

We identified 642 unique patients evaluated in the retina clinic with a diagnosis of DM type II between December 31, 2019, and December 31, 2020. Patients were unique based on MRN. We then applied exclusion criteria including if patients had no documented ophthalmology follow-up or if they were missing documentation regarding recent A1c values. Additionally, patients less than 55 years of age were excluded from the study as primary AMD is rare in a younger cohort of patients. 

Statistical methods

To compare demographic and clinical differences between patients with a diagnosis of AMD and those without, the cohort was then split into two groups based on the presence of a diagnosis of AMD. Differences in demographic characteristics between patients with and without a diagnosis of AMD were tested using the Wilcoxon rank-sum test for continuous variables and Fisher’s exact test for binary variables. We report medians with 25th and 75th percentiles for continuous variables and means with percentages for binary variables. Differences between groups were considered significant at p<0.007 by correcting for multiple comparisons using Bonferroni correction, and all statistical tests were two-sided.

A multivariable logistic regression model was created to identify factors associated with a diagnosis of AMD. Variables included in the regression model were age, sex, provider seen, presence of past or active smoking, presence of a diagnosis of DR (any stage), and most recent A1c value (within three months of the appointment). To determine whether a certain level of diabetic control was associated with a diagnosis of AMD (for example, A1c>9.0), logistic regression was repeated using a series of cutoff values for A1c including A1c>7.0, A1c>8.0, A1c>9.0, and A1c>10.0. For the multivariate regression analyses, differences were considered statistically significant at p<0.05. Stata version 14 was used for all analyses.

## Results

**Figure 1:**
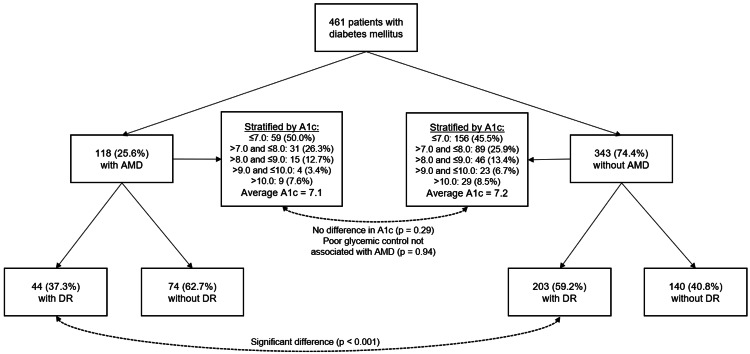
Flow chart summarizing methods and observed relationships between AMD, DR, and A1c DR, diabetic retinopathy; AMD, age-related macular degeneration

Between 12/31/2019 and 12/31/2020, 642 unique patients were seen in our retina clinic with a diagnosis of DM type II. After applying the exclusion criteria, 461 patients remained for analysis. The baseline characteristics of this group are outlined in Table 2, including a comparison of the characteristics of those with and without a diagnosis of AMD. Out of the 461 patients, 118 (25.6%) had a diagnosis of AMD. Compared to the control group, patients with AMD were found to be significantly older on average (66 compared to 69 years old, respectively; p=0.005), and significantly less likely to have a diagnosis of DR (59.18% compared to 37.29%, respectively; p<0.001) (Table 2). These differences between groups remain statistically significant when correcting for multiple comparisons. A1c values were not different between groups (p=0.29) (Table 2). A visual summary of methods and observed relationships between AMD, DR, and A1c is depicted in Figure 1.

**Table 2:** Demographics of patients seen at Long Island retina clinic over 55 years of age with and without AMD *Statistically significant with p<0.007 N, total number of patients; n, number of patients within each group; DR, diabetic retinopathy; AMD, age-related macular degeneration

Total cohort				AMD=Yes			AMD=No			
Variable	N	n	%	N	n	%	N	n	%	P-value
Age*	461	67 (61-73)		118	69 (62-76)		343	66 (61-72)		0.005
Female	461	210	45.55%	118	54	45.76%	343	156	45.48%	1.00
Smoker	461	145	31.45%	118	32	27.12%	343	113	32.94%	0.25
Provider	461	134	29.07%	118	31	26.27%	343	103	30.03%	0.48
DR*	461	247	53.58%	118	44	37.29%	343	203	59.18%	<0.001
Hemoglobin A1c	461	7.2 (6.5-8.2)		118	7.05 (6.5-8)		343	7.2 (6.5-8.3)		0.29

**Figure 2:**
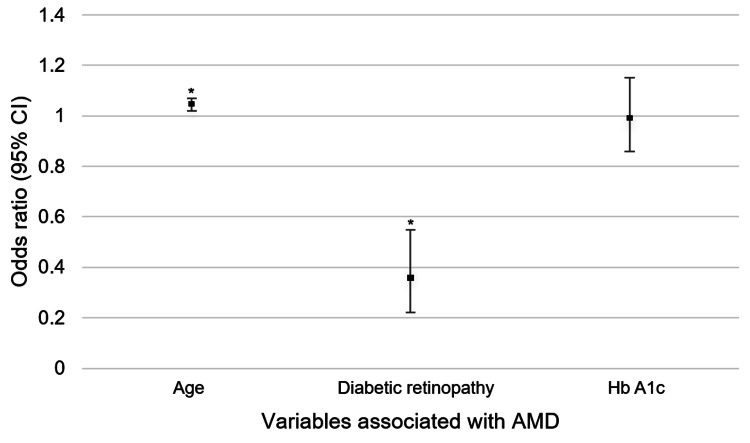
Variables associated with AMD Error bars represent 95% CI. *Statistically significant with p<0.05 AMD, age-related macular degeneration

Multiple logistic regression analyses were performed to determine associations between patient factors and AMD. Increased age was found to be significantly associated with AMD diagnosis (OR 1.05; p=0.001) (Table 3, Figure 2). DR in this cohort was significantly negatively associated with AMD (OR 0.35; p<0.001) (Table 3, Figure 2). No significant relationship was found between AMD and A1c values (Table 3, Figure 2).

**Table 3:** Variables significantly associated with AMD after multiple regression analysis *Statistically significant with p<0.05 DR, diabetic retinopathy; AMD, age-related macular degeneration

Factor	Odds ratio	Standard error	P-value	95% CI
Age*	1.05	0.01	0.001	1.02-1.07
DR*	0.35	0.08	0.000	0.22-0.55
Hemoglobin A1c	0.99	0.07	0.939	0.86-1.15

## Discussion

This study explored the association between AMD and DR while controlling for hemoglobin A1c. In our study population of diabetic patients seen for retina evaluation, we found that the presence of AMD of any degree was associated with significantly reduced odds of having a diagnosis of DR (OR 0.35). This finding was surprising given that there was no average difference in A1c values between the AMD and non-AMD cohorts. Furthermore, our AMD cohort was older on average than those without an AMD diagnosis and still displayed significantly reduced rates of DR, even though DR increases in incidence with age [18]. To ensure our statistical methods were sound, we ran an additional regression that confirmed that elevations in A1c were associated positively with a diagnosis of any DR (OR 1.20). 

In the literature, the association between AMD and DR is inconsistent. Our findings that DR is associated with reduced odds of having AMD agree with those of several recent studies [13,19-21]. In the clinical context, these findings may suggest a less aggressive DR screening or management approach in those with AMD and diabetes. However, other studies suggest that DR and DM are positive risk factors for the development of AMD, with an odds ratio as high as 2.22 [12,22,23]. Our study is unique in that we take into account glycemic control for a more nuanced analysis.

Given the uncertainty that persists in the literature, to date there is insufficient data to recommend such changes to screening protocols, as reducing screening may result in harm. On the other hand, if DR were, as some studies have suggested, a risk factor for AMD, patients with DR may benefit from more frequent AMD screening. Furthermore, physicians may consider early counseling on AMD prevention and recommend preventative measures such as smoking cessation and initiation of AREDS supplements for patients at risk.

As these studies were conducted in different patient populations internationally, such as Greece, South India, Taiwan, and the US, one explanation for these different results could be the difference in population health. Studies have shown that both personal-level and area-level socio-economic factors such as education and income affect the prevalence of both AMD and DR [24-26]. Moreover, variations in lifestyle factors, such as diet, physical activity, and exposure to environmental risk factors, may contribute to discrepancies in disease prevalence and progression across populations. Similarly, the prevalence rates of AMD and DR vary across different regions of the world. For example, Europeans have a higher rate of AMD than Asians, and DR disproportionately affects those in Africa and North America than in other continents [1,27]. These findings emphasize the potential interaction of genetics, environment, socio-economic factors, and regional differences in AMD and DR disease patterns.

Along with glycemic control, other factors are known to play a role in the pathogenesis of DR including duration of diabetes, ethnicity, hypertension, and smoking status [28,29]. An association between AMD and DR is possible given the shared risk factors such as obesity, age, dyslipidemia, and a common VEGF pathway thought to contribute to blood-retinal barrier breakdown [30]. All these factors could contribute to a relationship between AMD and DR, although they do not explain how different this relationship appears to be across populations. Therefore, given the complex, likely multivariate, relationship that exists between AMD and DR, researchers should be cautious when using DR as a surrogate for diabetic control in the context of AMD. 

Rather than strongly recommending for or against modifications in screening protocols and clinical practice, our study contributes to the literature with another set of analyses that will spark further investigation. Unlike other studies, here we control for A1c in our analysis to provide a more complex understanding that AMD may be associated with significantly reduced odds of DR, despite no difference in A1c between AMD and non-AMD groups. As such, we demonstrate that other factors beyond glycemic control, such as environmental risk factors, lifestyle factors, and duration of diabetes as discussed previously, likely modulate this relationship. Researchers must continue to explore the interaction between AMD and DR among different populations to see whether population demographics or other comorbidities may be responsible for this complex association. 

Regardless, physicians should inform patients diagnosed with either AMD or DR of the unclear relationship between the two conditions. They must emphasize the importance of regular follow-ups to monitor disease progression or development; they must emphasize addressing modifiable risk factors, including sugar intake, tobacco use, and hypertension, to minimize future consequences.

Limitations

​​We recognize several limitations to the study performed. In our analysis, we showed that the hemoglobin A1c value did not alter the relationship we were investigating between DR and the development of AMD. While the sample size utilized in our study exceeded that required as determined by our initial power analyses, future chart reviews involving a larger sample size would be useful. Another limitation of our study that impairs our ability to compare to previous studies was our inability to calculate a CCI due to data collection limitations. This index can be used to compare relative illness burden between populations analyzed and may have helped to elucidate differences between populations. Future studies should ensure they are able to calculate this index to allow for comparison since these associations seem to vary greatly with different study populations.

Other limitations include a reliance on diagnosis codes when identifying our study population. While diagnosis codes help to identify and stratify patients with relative ease, they do not capture the full clinical picture: treatment status, disease progression, comorbidities, and frequency of follow-up are not taken into account. In addition, physician-to-physician differences in diagnostic thresholds, particularly for classifying mild DR or AMD, can vary across institutions or practices, where slightly different criteria or grading systems may be applied. Together, these areas of variability may skew patient selection and limit the generalizability of our findings.

Additionally, subset data including degree of AMD and degree of DR were also not analyzed in this study. Perhaps some of the relationships between AMD and DR depend on disease severity. Future studies with more in-depth analysis are needed. Finally, a potential limitation exists considering that our study has a cross-sectional design. In taking this approach, we did not follow patients to determine if they would develop AMD but rather assessed this association at one point in time. By assessing only one time point, the study was unable to assess for a temporal association that exists between AMD and DR development. As such, future studies should aim to assess this temporal relationship through randomized control trials.

## Conclusions

In conclusion, our study controlled for glycemic index in examining the association between AMD and DR in diabetic patients. We found that a diagnosis of AMD significantly reduced the likelihood of DR diagnosis. This outcome held true independent of A1c levels and age. Despite limitations such as the reliance on diagnosis codes, a limited sample size, and a cross-sectional design, our findings contribute to the current literature on the complex relationship between AMD and DR. Future research should address these limitations, potentially through longitudinal studies, to better explore the causal and temporal dynamics between these conditions. Continuing to investigate AMD and DR across different populations may clarify the genetic, environmental, and socioeconomic factors of these diseases and help guide treatment strategies. 
